# Metabolic syndrome predicts vascular changes in whole body magnetic resonance imaging in patients with long standing diabetes mellitus

**DOI:** 10.1186/1475-2840-9-44

**Published:** 2010-08-30

**Authors:** Hannes M Findeisen, Sabine Weckbach, Renée G Stark, Maximilian F Reiser, Stefan O Schoenberg, Klaus G Parhofer

**Affiliations:** 1 Department of Internal Medicine II, University Hospitals - Grosshadern, Ludwig-Maximilians-University Munich, Munich, Germany; 2 Department of Clinical Radiology, University Hospitals - Grosshadern, Ludwig-Maximilians-University Munich, Munich, Germany; 3 Helmholtz Zentrum, Research Center for Environmental Health, Neuherberg, Germany; 4 Department of Clinical Radiology and Nuclear Medicine, University Hospital Mannheim, University of Heidelberg, Mannheim, Germany

## Abstract

**Background:**

Although diabetic patients have an increased rate of cardio-vascular events, there is considerable heterogeneity with respect to cardiovascular risk, requiring new approaches to individual cardiovascular risk factor assessment. In this study we used whole body-MR-angiography (WB-MRA) to assess the degree of atherosclerosis in patients with long-standing diabetes and to determine the association between metabolic syndrome (MetS) and atherosclerotic burden.

**Methods:**

Long standing (≥10 years) type 1 and type 2 diabetic patients (n = 59; 31 males; 63.3 ± 1.7 years) were examined by WB-MRA. Based on the findings in each vessel, we developed an overall score representing the patient's vascular atherosclerotic burden (MRI-score). The score's association with components of the MetS was assessed.

**Results:**

The median MRI-score was 1.18 [range: 1.00-2.41] and MetS was present in 58% of the cohort (type 2 diabetics: 73%; type 1 diabetics: 26%). Age (p = 0.0002), HDL-cholesterol (p = 0.016), hypertension (p = 0.0008), nephropathy (p = 0.0093), CHD (p = 0.001) and MetS (p = 0.0011) were significantly associated with the score. Adjusted for age and sex, the score was significantly (p = 0.02) higher in diabetics with MetS (1.450 [1.328-1.572]) compared to those without MetS (1.108 [0.966-1.50]). The number of MetS components was associated with a linear increase in the MRI-score (increase in score: 0.09/MetS component; r^2 ^= 0.24, p = 0.038). Finally, using an established risk algorithm, we found a significant association between MRI-score and 10-year risk for CHD, fatal CHD and stroke.

**Conclusion:**

In this high-risk diabetic population, WB-MRA revealed large heterogeneity in the degree of systemic atherosclerosis. Presence and number of traits of the MetS are associated with the extent of atherosclerotic burden. These results support the perspective that diabetic patients are a heterogeneous population with increased but varying prevalence of atherosclerosis and risk.

## Introduction

Today diabetes mellitus is often classified as a coronary heart disease (CHD) equivalent [1], which has significantly influenced the guidelines for the treatment of diabetic complications over the last couple of years [2]. According to the American Diabetes Association (ADA), all diabetic patients above 40 years of age should be managed aggressively regarding risk factors with lipid lowering and antiplatelet therapy, similar to patients with a history of myocardial infarction [3]. Although the term CHD equivalent focused attention on the increased cardiovascular risk of diabetic patients, with coronary heart disease being the leading cause of mortality among people with diabetes [4], it is also obvious that not all patients have the same risk [2,5,6]. Individualized risk assessment could therefore lead to more appropriate risk factor interventions [2,7]. Extensive research has focused on the metabolic syndrome (MetS), a cluster of cardiovascular risk factors consisting of visceral obesity, hyperglycemia, increased plasma triglycerides, decreased HDL-cholesterol and hypertension, as a possible marker for increased cardiovascular risk in diabetic patients [8]. Many studies have in fact found an association between cardiovascular risk and MetS in type 2 [8-10] and some also in type 1 diabetes [11,12], yet others have found conflicting results in both types of diabetes [13,14].

Hence the MetS has become the center of an intensive debate due to different and changing definitions, lack of a unifying pathophysiological concept, disputed predictive value regarding CHD and uncertain specific therapeutic consequences [8,15,16]. While the joint statement from the American Diabetes Association and the European Association for the Study of Diabetes (EASD) stated that the term MetS should not be used as a diagnosis and that diabetes should be excluded from the definition of the MetS since the presence of a MetS seems to provide no additional predictive value for cardiovascular complications or therapeutic consequences in patients with diabetes [8], other still find the MetS useful particularly for clinical purposes [17-20].

Previously we have used whole-body magnetic resonance angiography (WB-MRA) in patients with long-standing type 1 or type 2 diabetes to obtain comprehensive vascular imaging (except coronaries) [21]. In our study we found a high prevalence of occult atherosclerotic disease, demonstrating that the true extent of atherosclerotic complications was largely underestimated in these patients [21]. Furthermore, we developed a score based on the WB-MRA findings representing the atherosclerotic burden of each patient. In the current study we have analyzed this data to address the following questions: 1. What is the variation of atherosclerotic burden in patients with long-standing diabetes? 2. Is the MetS associated with a high atherosclerotic score in these patients? 3. Is the atherosclerosis detected by WB-MRA associated with CHD and cardiovascular risk, estimated with the United Kingdom Prospective Diabetes Study (UKPDS) risk engine, an established risk algorithm for diabetic patients?

## Methods

### Study population

The original study included 65 patients with type 1 (T1DM) or type 2 (T2DM) diabetes of more than 10 years which was published previously [21]. The parameters analyzed in this study were available for 59 patients of this population (28 females, 31 males). The patients were enrolled over a 16 month period in our Medical Department. The study was approved by the ethics committee and all patients provided written consent prior to their inclusion.

Inclusion criteria were diagnosis of type 1 or type 2 diabetes mellitus of >10 years duration. Exclusion criteria were pregnancy, allergic reaction to Gadolinium-chelates and a creatinine clearance <30 ml/min per 1.73 m^2 ^to avoid nephrogenic systemic fibrosis (NSF) [22]. Creatinine clearance was assessed using the Cockroft-Gault formula.

At the time of inclusion, a number of parameters were registered including age, sex, type and duration of diabetes, smoking history, body mass index (BMI), blood pressure, HbA1c, triglycerides, low density lipoprotein (LDL)-cholesterol, high density lipoprotein (HDL)-cholesterol and creatinine. CHD status was assessed, defined as documented history of myocardial infarction, stenosis of >50% on coronary angiogram, coronary intervention or bypass grafting. All patients received the whole body MRI in the Department of Radiology within 3 months after recording the above mentioned parameters.

The MetS was identified according to the definition of the American Heart Association and the National Heart, Lung, and Blood Institute (AHA/NHBLI) [17]: triglycerides ≥150 mg/dl; HDL-cholesterol <40 mg/dl (male) or <50 mg/dl (female); systolic blood pressure ≥130 mmHg or diastolic blood pressure ≥85 mmHg or anti-hypertensive medication, with the exception that BMI (>30 kg/m^2^) was used instead of waist circumference. MetS was diagnosed when in addition to diabetes two or more of these traits were present.

### MRA

MR imaging studies were performed as described previously on a 1.5 T and 3 T whole body MR system (Magnetom Avanto and Magnetom Tim Trio, Siemens Medical Solutions, Erlangen, Germany) equipped with 32 receiver channels [21]. Twelve patients were scanned at 1.5 T, 47 at 3 T after installation of the new MR scanner. In brief, IPAT (integrated parallel acquisition techniques) and a GRAPPA (generalized autocalibrating partially parallel acquisitions) reconstruction algorithm were used with acceleration factors between 2 and 3. Various combinations of head and neck coils, spine array coils and various body coils were employed (Siemens Medical Solutions, Erlangen, Germany). All patients received an intravenous injection of Omniscan^® ^(Gadodiamide, GE Healthcare).

First a time-of-flight (TOF)-MR angiogram of the cerebral arteries with a spatial resolution of 0.7 × 0.5 × 0.7 mm was acquired. Then 3D-Gd-MR-angiography of the carotids (resolution 1.0 × 1.0 × 1.0, iPAT factor 3), the abdominal aorta (1.4 × 1.1 × 1.2, iPAT factor 3), the thighs (1.1 × 1.1 × 1.1, iPAT factor 2), the calves and pedal arteries (1.0 × 1.0 × 1.0, iPAT factor 2) was obtained. The same scanning parameters and spatial resolutions were used at 1.5 T and 3 T. A time-resolved CE-sequence of the lower calf and pedal arteries (1.4 × 1.4 × 1.5, iPAT factor 3, temporal resolution 3.7 s/frame) was performed after repositioning the patient in order to compensate for a shortened range of table movement in the Magnetom Trio and to increase diagnostic accuracy.

The MR exams of the diabetic group were evaluated by two experienced radiologists who were blinded to all clinical information, in a consensus reading. The cases were presented in groups of five cases per reading session in randomized order over a time period of four weeks. MRA data sets were evaluated by multiplanar reformats (MPR) and small volume maximum intensity projections (thin MIP) for detection of stenosis as well as volume-rendering (INSPACE) for detection of anatomic variants. There were 22 segments analyzed and all but the abdominal aorta and circle of Willis were bilateral [23]. They included: 1) common carotid artery, 2) internal extracerebral carotid artery, 3) intracerebral carotid artery/circle of Willis, 4) vertebral artery, 5) abdominal aorta, 6) renal artery, 7) common iliac/femoral artery, 8) internal iliac artery, 9) superficial femoral and popliteal artery, 10) anterior tibial artery, 11) posterior tibial artery and 12) peroneal artery.

As previously published we have created a new scoring system for the degree of atherosclerotic disease burden found in the diabetic patients, entitled the "vessel score" [21]. The vessel score is determined as follows: atherosclerotic lesions were quantified in each vessel by a grading scheme using 6 levels ranging from normal to occlusion with grade 1 = normal vessel without visible changes or only mild wall irregularities, grade 2 = non significant stenosis, grade 3 = singular significant stenosis exceeding 50% of diameter, grade 4 = multi-segmental significant stenoses with at least one exceeding 50% of diameter, grade 5 = fading vessel with incomplete visualization over the entire segment, grade 6 = complete occlusion with (a) or without (b) reconstitution. The sum of grades of all evaluated vessels divided by the number of vessels resulted in an average vessel score (average grade per evaluated vessel) representing the patient's vascular status.

### Statistics

Continuous variables were described with mean ± standard deviation or median and range. Chi-squared tests were used to test for simple associations between dichotomous variables. Correlation between continuous variables was tested with the Spearman rank correlation. Differences between the mean were tested with a t-test. A general linear model (GLM) was used to test for associations between the score and other variables after adjustment for age and sex. A p-value of <0.05 indicated significance. SAS 9.1 was used for statistical evaluation.

## Results

**Figure 1:**
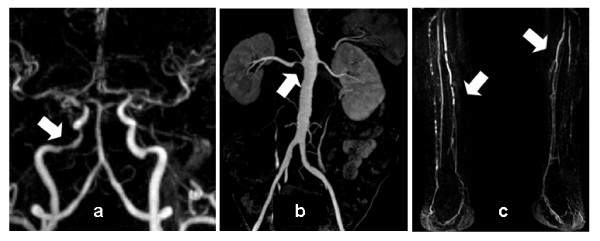
**MRA demonstrating typical atherosclerotic findings in patients with longstanding diabetes**. A: moderate stenosis of the right middle cerebral artery, B: severe right renal artery stenosis, C: advanced multisegmental bilateral peripheral artery disease.

Fifty nine patients (31 males, 63 ± 13 years) with long standing (>10 years, mean 20.7 years) type 1 (n = 19) or type 2 (n = 40) diabetes were examined by MRA (Table 1). Prevalence rates of vascular lesions were 46% for peripheral artery disease, 20% for cerebrovascular disease and 8% for renal artery stenosis. The observed changes are described in more detail in reference [21]. Typical findings of vascular lesions are presented in figure 1. The range of the MRI-score was 1.00 to 2.41 with an average score of 1.31 and a median of 1.18. In 22 patients the score was 1.00, indicating that these patients had at most wall irregularities in all vessels (Table 2).

**Table 1:** Study Population

	Total	Men	Women
**Type 1 Diabetes mellitus**	19 (32%)	11 (35%)	8 (29%)
**Type 2 Diabetes mellitus**	40 (68%)	20 (65%)	20 (71%)
**Age**	63.3 ± 1.7	63.5 ± 2.4	63 ± 2.6
**Diabetes duration**	20.7 ± 1.4	20.4 ± 2.1	21 ± 1.7
**Hypertension**	51 (86%)	27 (87%)	22 (79%)
**Systolic blood pressure**	135 ± 2	133 ± 3	137 ± 3
**Diastolic blood pressure**	77 ± 1	76 ± 2	77 ± 2
**Hyperlipoproteinemia**	39 (66%)	19 (61%)	20 (71%)
**Smoker**	4 (7%)	3 (10%)	1 (4%)
**BMI (kg/m^2^)**	27.8 ± 0.6	27.3 ± 0.6	28.3 ± 1.2
**Coronary heart disease**	22 (37%)	15 (48%)	7 (25%)
**Cerebrovascular disease**	11 (19%)	8 (26%)	3 (11%)
**Peripheral artery disease**	15 (25%)	10 (32%)	5 (18%)
**Insulintherapy**	51 (86%)	25 (81%)	26 (93%)
**Oral antidiabetic drugs**	21 (36%)	11 (35%)	10 (36%)
**ACEI/ARB**	43 (73%)	23 (74%)	20 (71%)
**Lipid lowering drugs**	36 (61%)	19 (61%)	17 (61%)
**HbA1c (%)**	7.3 ± 0.1	7.5 ± 0.2	7.1 ± 1.3
**Creatinine (mg/dl)**	1.2 ± 0.2	1.3 ± 0.2	1.1 ± 0.2*
**LDL-Cholesterol (mg/dl)**	101.9 ± 3.7	102.4 ± 5.1	101.3 ± 5.6
**HDL-Cholesterol (mg/dl)**	49.9 ± 1.9	47.2 ± 2.6	52.6 ± 2.8
**Triglycerides (mg/dl)**	181.0 ± 16.5	168.3 ± 18.3	195.1 ± 29.0

Parameters without a unit in brackets indicate number of patients (and percentages).

Parameters with a unit in brackets are mean ± SEM

ACEI, angiotensin-converting enzyme inhibitors; ARB, angiotensin receptor blocker

LDL, low density lipoprotein; HDL, high density lipoprotein

* p < 0.05 vs. men

**Table 2:** Distribution of score

Score	Total	Men	Women
1.00	22	9	13
1.01-1.50	23	12	11
1.51-2.00	9	7	2
>2.00	5	3	2

In an unadjusted analysis, age, hypertension, low HDL-cholesterol, MetS, CHD status and nephropathy were significantly associated with the score, while neither sex, diabetes duration, BMI, blood pressure, triglycerides, LDL-cholesterol, creatinine, HbA1c, retinopathy, nephropathy or neuropathy were associated with the score (Table 3).

**Table 3:** Unadjusted association with score

	p-value
Age^a^	**0.0002**
Sex^b^	0.1082
Diabetes duration^a^	0.9434
Hypertension^b^	**0.0008**
Systolic BP^a^	0.2233
Diastolic BP^a^	0.731
LDL-cholesterol^a^	0.2951
HDL-cholesterol^a^	**0.016**
Triglycerides^a^	0.0535
BMI^a^	0.826
HbA1c^a^	0.4529
MetS^b^	**0.0011**
Creatinine^a^	0.0601
CHD^b^	**0.0001**
Retinopathy^b^	0.0689
Neuropathy^b^	0.6291
Nephropathy^b^	0.0093

BP: blood pressure; CHD: coronary heart disease; MetS: metabolic syndrome; ^a ^p-value refers to correlation coefficient; ^b ^p-value refers to Wilcoxon test;

MetS was present in 58% (n = 34) of the patients (T2DM: 73%, n = 29; T1DM: 26%, n = 5) with no sex difference. Patients without MetS were considerably younger (56 ± 16 yr vs. 69 ± 8 yr). All patients with the MetS had hypertension or anti-hypertensive medication due to diagnosed hypertension, 26 had high triglycerides, 22 had low HDL-cholesterol and 14 patients had a BMI >30 kg/m^2^. 14 Patients had 2 traits of the MetS, 12 had 3 and 8 had all 4 traits. 18 Patients without MetS still had 1 trait of the MetS, most often hypertension (n = 15).

**Figure 2:**
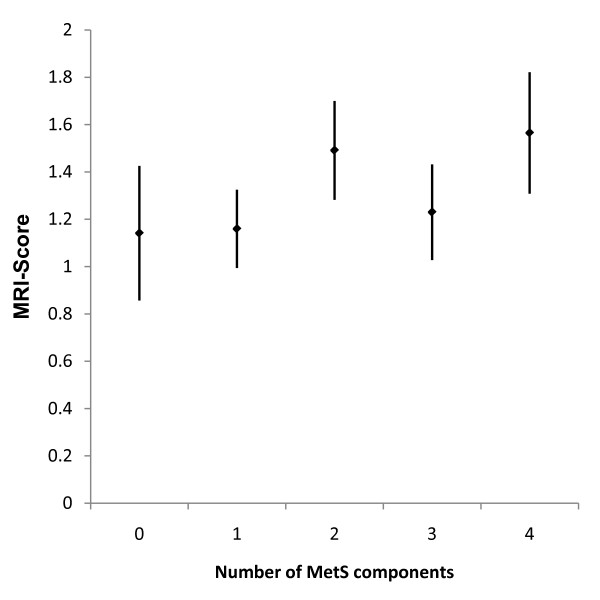
**Number of components of the MetS is associated with an increase in MRI-score (increase in score: 0.09/MetS component; r^2 ^= 0.24, p = 0.038 adjusted for age and sex)**. Data is presented as mean ± SEM.

Patients with MetS had a higher mean score than those without MetS (1.450 [1.328-1.572] vs. 1.108 [0.966-1.250] respectively, p = 0.0002). This association remained significant (p = 0.02) after adjustment for age and sex. Furthermore, we found a dose-response relationship between the number of MetS components and the MRA-score (increase in score: 0.09/MetS component; r^2 ^= 0.24, p = 0.038 adjusted for age and sex, figure 2). Similar results were observed in the subgroup of T2DM. After adjustment for age and sex T2DM patients with MetS had a significantly higher mean score than those without MetS (1.438 [1.297-1.580] vs. 1.130 [0.900-1.361] respectively, p = 0.03). In contrast, in T1DM subjects, the score was only associated with the presence of MetS in an unadjusted model. After adjustment for age and sex the score was similar in T1DM patients with and without MetS.

To further validate the MRA-score, we analyzed the association of the score with cardiovascular risk calculations in T2DM patients, using the United Kingdom Prospective Diabetes Study (UKPDS) risk engine [24]. Consistent with the significant association of score and previously diagnosed CHD, a high score was significantly associated with a high 10-year risk for CHD, fatal CHD and stroke (Table 4).

**Table 4:** Correlation between MRA-score and cardiovascular risk (UKPDS risk engine)

	r	p-value
CHD risk	0.46	**0.0025**
Fatal CHD risk	0.45	**0.0036**
Stroke risk	0.37	**0.0204**
Fatal stroke risk	0.29	0.073

r, Spearman correlation coefficient

## Discussion

During the last 10 years the classification of diabetes as a CHD equivalent has helped to improve standards of diabetes care by underlining the importance of cardiovascular risk factor management [3]. However, it is also obvious that this simplified concept falls short of adequately describing the true cardiovascular risk of a highly heterogeneous population [5,6]. The presence of the MetS, a cluster of cardiovascular risk factors, may help to refine risk factor assessment and treatment in diabetic patients. In this study, 59 patients with long standing diabetes mellitus underwent whole body MRA with quantification of atherosclerosis by determining an overall atherosclerosis score. Using this score we investigated the degree of variation in macrovascular disease and found a significant association between the score and the presence of the MetS as well as a dose-response relationship between the score and the number of MetS-components.

Despite the general high risk of the population with an average diabetes duration of 20.7 years and additional cardiovascular risk factors in many patients, we found very pronounced differences in the degree of atherosclerotic burden. The WB-MRA based score ranged from 1.00 to 2.41 with a median of 1.18. More than one third of the population (n = 22) had a score value of 1.00, indicating that these patients had no atherosclerotic changes or at most wall irregularities in all scanned vessels. In contrast, all patients with a score above 2.0 (n = 5) had at least 3 completely occluded vessels and several other stenotic lesions. These results are in line with the perspective of diabetic patients as a heterogeneous population with increased but variable prevalence of atherosclerosis and cardiovascular events [25,26].

In our study population a high score was significantly associated with age, HDL-cholesterol, MetS and CHD status. The number of components of the MetS was associated with a linear increase in the MRI-score. Furthermore, consistent with previous reports evaluating MRI-based atherosclerosis scores [27,28], a high score was significantly associated with estimated cardiovascular risk using an established risk algorithm.

Despite significant progress in recent years with respect to risk factor modification particularly through the use of lipid lowering agents and drugs targeting the renin-angiotensin system [29], several studies have found that there is still a large gap between guidelines and clinical practice [30,31]. Furthermore, recent landmark studies aimed at investigating an even more aggressive treatment of hyperglycemia, blood pressure and dyslipidemia failed to show additional benefits in diabetic patients [32,33]. A more individualized risk assessment could hence allow clinicians to apply flexible treatment goals to their diabetic patients and help to focus their attention and resources on the patients most at risk among the diabetic population [34]. Including the well known concept of MetS into cardiovascular risk assessment and treatment decisions may help to individualize and further improve diabetes care.

There are several limitations to our study. First the prognostic significance of the detected vascular lesions is uncertain, due to the lack of follow up data. The association with the UKPDS risk calculations also must be interpreted with caution since the risk engine is not validated for a diabetes duration of >20 years and has an uncertain validity for patients with established cardiovascular disease, although only patients with acute cardiovascular disease were excluded in the original UPKDS study [35]. Furthermore, there was no examination of the coronary arteries. However, cerebrovascular disease and peripheral artery disease are both well established predictors of cardiovascular mortality [26] and also in this study, the score showed a strong association with CHD status. Other limitations of this study are the relatively small sample size and single on treatment measurements. These factors limit the significance of the statistical associations. Another limitation relates to the scoring system. First of all, changes in all arteries were treated equally. Thus, there was no "weighting" of arteries (for example: changes of fibular arteries were considered as relevant as those of carotid arteries). From a clinical point of view, changes in some vascular beds are obviously more relevant than changes in others. However, a correct weighting of arteries is not possible. Furthermore, the grading of the changes in the individual arteries was considered to be linear, although this is not the case. For example: "multi-segmental significant stenoses with at least one exceeding 50% of the vessel diameter" (grading 4) was considered to represent double the amount of atherosclerosis as "non significant stenosis" (grading 2). However, in order to create an overall score for each patient, location and severity of changes had to be described numerically.

## Conclusion

In conclusion our results contribute further evidence to the perspective of diabetic patients as a heterogeneous population with increased but varying prevalence of cardiovascular disease and risk. In our study, presence of the Metabolic Syndrome and the number of its traits helped to identify those patients with the largest atherosclerotic burden. Further research is needed to improve the characterization of risk in diabetic patients and to adapt the treatment guidelines to different risk classes.

## Competing interests

The authors declare that they have no competing interests.

## Authors' contributions

HMF recruited study participants, collected patient history, performed data analysis, and drafted the manuscript. SW and SOS performed MRA data analysis and participated in designing the study, and critically revising the manuscript. RS performed the statistical analysis. MR participated in designing the study and revising the manuscript. KGP participated in designing the study, data analysis, performing the statistical analysis, and drafting the manuscript. All authors read and approved the final manuscript.
